# Improving the Orbit Injection Accuracy of a Launch Vehicle by Using a Mode-Switching Strategy for a Dual-Axis Rotational Inertial Navigation System

**DOI:** 10.3390/s25010177

**Published:** 2024-12-31

**Authors:** Jun Li, Shifeng Zhang, Zhenlin Ma

**Affiliations:** 1 College of Aerospace and Engineering, National University of Defense Technology, Changsha 410073, China; lijun17a@nudt.edu.cn (J.L.); mazhenlin23@nudt.edu.cn (Z.M.); 2 Hunan Provincial Key Laboratory of Aerospace Cross-Domain Flight Vehicle System and Control Technology, Changsha 410073, China

**Keywords:** rotational inertial navigation system, orbit injection accuracy, mode-switching strategy

## Abstract

Due to a short flight time, the dual-axis rotational inertial navigation system carried by some launch vehicles or missiles is often only used for self-calibration and self-alignment. It is generally in the strap-down state rather than the rotation modulation state during flight. This wastes the precision potential of the navigation system. Therefore, a mode-switching strategy is proposed in this paper to improve the orbit injection accuracy of a launch vehicle. The rotational navigation system of the launch vehicle is in the near-platform mode or strap-down mode at different periods. It is switched in a timely manner to take advantage of the various modes. Numerical simulation and turntable experiment results show that compared with the traditional strap-down inertial mode, the mode-switching strategy can effectively improve the orbit accuracy of the launch vehicle by about 12% to 13% and hardly increases any costs.

## 1. Introduction

An inertial sensor has error terms that cannot be calibrated thoroughly, which is the main factor that restricts the orbit injection accuracy of launch vehicles, ships, and underwater vehicles sailing for a long time [1,2]. To reduce navigation errors, in addition to the use of relatively mature inertial devices, a lot of research so far has focused on error compensation, including alignment and calibration [3,4,5,6] in advance and rotation modulation [7,8,9] under operation.

### 1.1. Related Work

Rotational inertial navigation systems (RINS) have been widely used in marine navigation, and corresponding alignment [10,11,12] and rotation schemes [13,14,15] have been studied extensively. Their principle is that the positive and negative signs of the navigation error change alternate through the periodic rotation of the frame axis so that there is an average of 0 in the modulation period. Generally speaking, the rotation modulation method is suitable for long-term and low-dynamic navigation. However, on high-dynamic platforms such as rockets and missiles, overload and rapid changes in angular velocity significantly decrease the effect of positive and negative cancellation. The research on rotational navigation systems under high-dynamic conditions, such as rockets, is mainly applied to high-speed spin bombs [16]. The inertial navigation system (INS) accuracy at high rotational speed is improved by single-axis rotation modulation after isolating the projectile’s angular velocity [17]. A novel rotational scheme is designed to compensate for the modulation angular rate instability at high spin [18,19]. This single-axis Rotation System is often called the rotation Semi-Strap-down Inertial Navigation System.

The use of rotational navigation systems for launch vehicles has yet to be thoroughly studied. Nevertheless, many of the inertial navigation systems on a spacecraft are equipped with a dual-axis rotating frame, fiber optic gyroscopes, and quartz accelerometers for self-calibration and self-alignment [20]. This kind of inertial navigation device is also known as the three-autonomy inertial navigation system [21] (autonomous calibration, autonomous alignment, and autonomous test). Although these systems have the capability of a dual-axis rotational inertial navigation system (an inertial device consisting of a 3-axis accelerometer and a 3-axis gyroscope mounted on two orthogonal rotating frames), they still adopt the strap-down mode instead of the rotation modulation mode in flight, mainly because the launch vehicle has a short flight time and it is difficult to run the integer multiple period modulation process. It is difficult for frame systems to achieve accurate modulation while isolating angular motion. However, given the rotational ability of these inertial navigation systems, it is a problem worth studying whether to configure appropriate navigation strategies to improve accuracy.

### 1.2. Our Work

**Figure 1:**
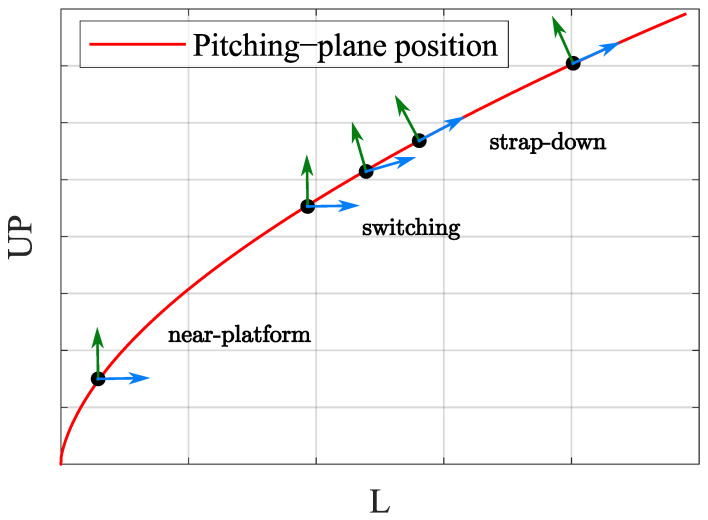
Simplified mode switch along the flight trajectory.

To solve this problem, this paper designs a new use scheme, that is, to make the inertial navigation system in the working state of strap-down or close to the near-platform in different periods during flight. According to the characteristics of flight trajectory, the navigation mode can be optimized at various time intervals, and the mode can be changed in time to improve the accuracy of navigation guidance. Specifically, we recommend that the INS be in quasi-platform mode for some time after the rocket launch, perform a mode switch during flight, lock the frame, and enter strap-down navigation mode. The entire process is shown in Figure 1. The switching time is determined by the numerical optimization method according to the error estimation theory established in this paper. This scheme does not need to change the structural design of the existing three-self inertial navigation system; it only needs to change its use mode so that the implementation cost is low.

This paper studies the concrete implementation scheme and gives the timing of mode switching based on sensitivity optimization. Finally, the effectiveness of the scheme is verified by semi-physical experiments for the rocket’s precise orbit injection.

The novelty of this paper mainly shows in the following aspects:

1. The error distribution model and simplified calculation method of the inertial navigation system with mode-switching are established;
2. The strategy of improving guidance accuracy by rotational inertial navigation mode switching is proposed and verified.

The subsequent content of the article is arranged as follows: Section 2 describes the inertial navigation error propagation model along flight trajectory and the mode switching concept. Section 3 describes the orbit injection accuracy estimation method for the launch vehicle and optimization-based mode-switching strategy. Section 4 describes numerical and semi-physical experiments to validate the proposed method. Section 6 summarizes the entire manuscript.

## 2. Problem Description

### 2.1. IMU Error Model

#### 2.1.1. Coordinate System

**Figure 2:**
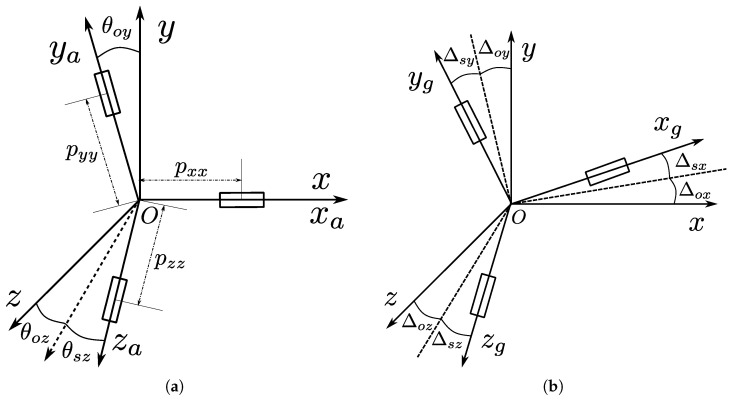
Measurement coordinate system. (**a**) Measurement coordinate system and acceleration-sensitive axis. (**b**) Measurement coordinate system and gyroscope-sensitive axis.

Figure 2 is the IMU coordinate system used in this paper, where $x_{a}$, $y_{a}$, $z_{a}$ are acceleration-sensitive axes. The measurement coordinate system is established relative to the acceleration assembly, the origin is at the reference center of the acceleration assembly, and the Ox axis is parallel to the sensitive axis of the *x* accelerometer. Oy is perpendicular to Ox in the plane spanned by the sensitive axes of the Ox and *y* accelerometers. Oz, $Ox_{m}$, and Oy form a right-handed coordinate system. Figure 2a shows the relationship between the acceleration-sensitive axis and the measurement coordinate system. Figure 2b shows the relationship between the gyro-sensitive axis and the measurement coordinate system.

#### 2.1.2. Gyro Error Model

The error model of the gyroscope with a quadratic term scaling coefficient is considered

(1) $$\tilde{\omega }=\omega +k_{g0}+k_{g1}\omega +k_{g2}\omega^{2}+\epsilon_{g}$$

where $\tilde{\omega }$ is the output angular velocity of the gyro assembly in units of rad/s. ω is the input angular velocity of the measurement coordinate system in units of rad/s.

(2) $$\tilde{\omega }=\left[\begin{array}{c} {\tilde{\omega }}_{x}\\ {\tilde{\omega }}_{y}\\ {\tilde{\omega }}_{z} \end{array}\right],\;\;\omega =\left[\begin{array}{c} \omega_{x}\\ \omega_{y}\\ \omega_{z} \end{array}\right],\;\;\omega^{2}=\left[\begin{array}{c} \omega_{x}^{2}\\ \omega_{y}^{2}\\ \omega_{z}^{2} \end{array}\right]$$

$k_{g0}$ is the bias of the gyro assembly in units of rad/s.

(3) $$k_{g0}=\left[\begin{array}{c} k_{g0x}\\ k_{g0y}\\ k_{g0z} \end{array}\right]$$

$k_{g1}$ is a dimensionless matrix composed of scale coefficient errors and installation errors of gyroscope assemblies.

(4) $$\begin{array}{cc} k_{g1} & =S_{g}N_{m}^{g}-I\\ & \approx \left[\begin{array}{ccc} 1+s_{gx} & & \\ & 1+s_{gy} & \\ & & 1+s_{gz} \end{array}\right]\left[\begin{array}{ccc} 1 & \Delta_{sx} & -\Delta_{ox}\\ -\Delta_{oy} & 1 & \Delta_{sy}\\ \Delta_{sz} & -\Delta_{mz} & 1 \end{array}\right]-I\\ & \approx \left[\begin{array}{ccc} s_{gx} & k_{gxy} & k_{gxz}\\ k_{gyx} & s_{gy} & k_{gyz}\\ k_{gzx} & k_{gzy} & s_{gz} \end{array}\right] \end{array}$$

Among them, $S_{g}$ is the dimensionless scale matrix, and $s_{gz}$ indicates the relative error of the scale coefficient. $N_{m}^{g}$ is the installation matrix. The installation error $\Delta_{**}$ is shown in Figure 2b.

$k_{\delta g2}$ is the error coefficient caused by the quadratic nonlinear change of the gyro scaling coefficient in units of s/rad.

(5) $$k_{\delta g2}=\left[\begin{array}{ccc} k_{\delta g2x} & & \\ & k_{\delta g2y} & \\ & & k_{\delta g2z} \end{array}\right]$$

$\epsilon_{g}$ indicates the random error of the gyro assembly in units of rad/s.

The gyro error model Equation (1) can be written as a matrix equation (Equation (6)).

(6) $$\tilde{\omega }-\omega =A_{g}\left(\omega \right)D_{g}+\epsilon_{g}$$

Among them

(7) $$\left\{\begin{array}{cc} A_{g}\left(\omega \right) & =\left[\begin{array}{ccc} I & {\mathrm{diag}}_{m}\left(\omega \right) & \mathrm{diag}(\omega .^{2}) \end{array}\right]\\ D_{g} & =\left[\begin{array}{c} k_{g0}\\ {\bar{k}}_{g1}\\ {\bar{k}}_{g2} \end{array}\right]\\ {\bar{k}}_{g1} & ={\left[s_{gx},\;k_{gxy},\;k_{gxz},\;k_{ayx},\;s_{gy},\;k_{gyz},\;k_{gzx},\;k_{gzy},\;s_{gz}\right]}^{T}\\ {\bar{k}}_{g2} & ={\left[k_{g2x},\;k_{g2y},\;k_{g2z}\right]}^{T} \end{array}\right.$$

The correlation diagonalization function is defined as follows:

(8) $$\left\{\begin{array}{cc} {\mathrm{diag}}_{m}\left(\omega \right) & =\left[\begin{array}{ccc} \omega^{T} & & \\ & \omega^{T} & \\ & & \omega^{T} \end{array}\right]\\ \mathrm{diag}\left(\omega .^{2}\right) & =\left[\begin{array}{ccc} \omega_{x}^{2} & & \\ & \omega_{y}^{2} & \\ & & \omega_{z}^{2} \end{array}\right] \end{array}\right.$$

#### 2.1.3. Accelerometer Error Model

The accelerometer error model including deviation, scaling coefficient, installation error, scaling coefficient asymmetry term, and quadratic term is considered in this paper.

(9) $$\tilde{f}=f+k_{a0}+k_{a1}f+k_{\delta a1}\left|f\right|+k_{a2}f^{2}+\epsilon_{a}$$

where $\tilde{f}$ is the output of the accelerometer assembly in units of [m/s^2^], f is the input apparent acceleration on the measurement coordinate system in units of [m/s^2^],

(10) $$\tilde{f}=\left[\begin{array}{c} {\tilde{f}}_{x}\\ {\tilde{f}}_{y}\\ {\tilde{f}}_{z} \end{array}\right],\;\;f=\left[\begin{array}{c} f_{x}\\ f_{y}\\ f_{z} \end{array}\right],\;\;f^{2}=\left[\begin{array}{c} f_{x}^{2}\\ f_{y}^{2}\\ f_{z}^{2} \end{array}\right]$$

$k_{a0}$ is the bias of the accelerometer assembly in units of [m/s^2^],

(11) $$k_{a0}=\left[\begin{array}{c} k_{a0x}\\ k_{a0y}\\ k_{a0z} \end{array}\right]$$

$k_{a1}$ is a dimensionless matrix composed of the scaling coefficient errors and installation errors of the accelerometer assemblies.

(12) $$\begin{array}{cc} k_{a1} & =S_{a}N_{m}^{a}-I\\ & \approx \left[\begin{array}{ccc} 1+s_{ax} & & \\ & 1+s_{ay} & \\ & & 1+s_{az} \end{array}\right]\left[\begin{array}{ccc} 1 & & \\ -\theta_{oy} & 1 & \\ \theta_{mz} & -\theta_{oz} & 1 \end{array}\right]-I\\ & \approx \left[\begin{array}{ccc} s_{ax} & & \\ k_{ayx} & s_{ay} & \\ k_{azx} & k_{azy} & s_{az} \end{array}\right] \end{array}$$

Among them, $S_{a}$ is the dimensionless scale matrix, and $s_{a*}$ indicates the relative error of the scale coefficient. $N_{m}^{a}$ is the installation matrix. The installation error $\theta_{**}$ is defined in Figure 2a.

$k_{\delta a1}$ is the dimensionless scale coefficient asymmetry term, and $k_{a2}$ is the scale coefficient quadratic term in units of [s^2^/m].

(13) $$\left\{\begin{array}{cc} k_{\delta a1} & =\left[\begin{array}{ccc} k_{\delta a1x} & & \\ & k_{\delta a1y} & \\ & & k_{\delta a1z} \end{array}\right]\\ k_{a2} & =\left[\begin{array}{ccc} k_{a2x} & & \\ & k_{a2y} & \\ & & k_{a2z} \end{array}\right] \end{array}\right.$$

The accelerometer error model Equation (9) can be written as a matrix of Equation (14).

(14) $$\tilde{f}-f=A_{a}\left(f\right)D_{a}+\epsilon_{a}$$

(15) $$\left\{\begin{array}{cc} A_{a}\left(f,\;\omega \right) & =\left[\begin{array}{cccc} I & {\mathrm{diag}}_{m}\left(f\right)I_{m} & \mathrm{diag}(|f|) & \mathrm{diag}(f.^{2}) \end{array}\right]\\ D_{a} & =\left[\begin{array}{c} k_{a0}\\ {\bar{k}}_{a1}\\ {\bar{k}}_{\delta a1}\\ {\bar{k}}_{a2} \end{array}\right]\\ {\bar{k}}_{a1} & ={\left[s_{ax},\;k_{ayx},\;s_{ay},\;k_{azx},\;k_{azy},\;s_{az}\right]}^{T}\\ {\bar{k}}_{\delta a1} & ={\left[k_{\delta a1x},\;k_{\delta a1y},\;k_{\delta a1z}\right]}^{T}\\ {\bar{k}}_{a2} & ={\left[k_{a2x},\;k_{a2y},\;k_{a2z}\right]}^{T} \end{array}\right.$$

The matrix $I_{m}$ is equal to $I_{9\times 9}$ and is obtained by removing columns 2, 3, and 6. And also,

(16) $$\left\{\begin{array}{cc} \mathrm{diag}\left(|f|\right) & =\left[\begin{array}{ccc} |f_{x}| & & \\ & |f_{y}| & \\ & & |f_{z}| \end{array}\right]\\ \mathrm{diag}\left(f.^{2}\right) & =\left[\begin{array}{ccc} f_{x}^{2} & & \\ & f_{y}^{2} & \\ & & f_{z}^{2} \end{array}\right] \end{array}\right.$$

### 2.2. Error Propagation Along the Flight Trajectory

The definition $r_{e}$ is the position of the vehicle in an Earth-centered, Earth-fixed coordinate system(ECEF), and $v_{e}=\frac{dr_{e}}{dt}$ is the velocity of the vehicle in ECEF.

In the Earth-centered inertial reference frame (ECI) *i*, we define $r^{i}$ and $v^{i}$ as follows:

(17) $$\left\{\begin{array}{cc} r^{i} & ≜C_{e}^{i}r^{e}\\ v^{i} & ≜C_{e}^{i}v^{e} \end{array}\right.$$

$C_{e}^{i}$ is the direction cosine matrix from the ECEF to the ECI.

It may be assumed that ECI and ECEF coincide at t=0; then,

(18) $$C_{e}^{i}=R_{z}\left(-\omega_{e}t\right)$$

where $R_{z}$ represents the direction cosine of the fundamental rotation about the *z* axis, and $\omega_{e}$ is the angular speed of the earth’s rotation, in units of [rad/s].

Then, the navigation equation in the geocentric inertial coordinate system can be expressed as

(19) $$\left\{\begin{array}{cc} {\dot{C}}_{m}^{i} & =C_{m}^{i}\left[\omega_{\mathrm{im}}^{m}\times \right]\\ {\dot{v}}^{i} & =C_{m}^{i}f^{m}+g^{i}-\left[\omega_{\mathrm{ie}}^{i}\times \right]v^{i}\\ {\dot{r}}^{i} & =v^{i}+\left[\omega_{\mathrm{ie}}^{i}\times \right]r^{i} \end{array}\right.$$

where $C_{m}^{i}$ is the direction cosine from the measurement coordinate system *m* to the geocentric inertial system *i*. $\omega_{\mathrm{im}}^{m}$ is the angular velocity of the measurement coordinate system *m* concerning the ECI *i*, projection on *m*, that is, ω in Equation (1). ${\dot{C}}_{m}^{i}$, ${\dot{v}}^{i}$, and ${\dot{r}}^{i}$, respectively, indicate a differential of $C_{m}^{i}$, $v^{i}$, and $r^{i}$ with respect to time. That is, Equation (19) is a first-order ordinary differential equation with respect to time. $\left[*\times \right]$ represents the antisymmetric matrix corresponding to the vector, defined as

(20) $$\left[x\times \right]=\left[\begin{array}{ccc} 0 & -x_{3} & x_{2}\\ x_{3} & 0 & -x_{1}\\ -x_{2} & x_{1} & 0 \end{array}\right],\;\mathrm{for}\;x=\left[\begin{array}{c} x_{1}\\ x_{2}\\ x_{3} \end{array}\right]$$

$f^{m}$ is f in equation Equation (9), and $\omega_{\mathrm{ie}}^{i}={\left[0,\;0,\;\omega_{e}\right]}^{T}$ is the angular velocity vector of the earth’s rotation. $g^{i}$ is the gravitational acceleration vector of the rotating earth in the ECI *i*, in units of $\left[\mathrm{kg}\cdot m/s^{2}\right]$, such that

(21) $$g^{i}=G^{i}-\left[\omega_{\mathrm{ie}}^{i}\times \right]\left[\omega_{\mathrm{ie}}^{i}\times \right]r^{i}$$

where $G^{i}$ is the gravitational acceleration in units of $\left[kg\cdot m/s^{2}\right]$, which is a function of position $r^{i}$; see ([22], Section 2.3.2).

By perturbing the navigation equation (Equation (19)) above, the error transfer equation can be obtained as

(22) $$\begin{array}{cc} \dot{\eta } & =C_{m}^{i}\delta \omega_{im}^{m} \end{array}$$

(23) $$\begin{array}{cc} \delta {\dot{v}}^{i} & =-\left[f^{i}\times \right]\eta +C_{m}^{i}\delta f^{m}-\left[\omega_{\mathrm{ie}}^{i}\times \right]\delta v^{i} \end{array}$$

(24) $$\begin{array}{cc} \delta {\dot{r}}^{i} & =\delta v^{i}+\left[\omega_{\mathrm{ie}}^{i}\times \right]\delta r^{i} \end{array}$$

where $f^{i}≜C_{m}^{i}f^{m}$. The existence of $\delta r^{i}$ actually causes an error in the gravity calculation $\delta g^{i}$. However, the error caused by this term is small for inertial navigation with high accuracy used by the launch vehicle, so it is ignored in the above formula.

Notice from the IMU error model Equations (6) and (14),

(25) $$\left\{\begin{array}{cc} \delta \omega_{im}^{m} & =A_{g}D_{g}+\epsilon_{g}\\ \delta f^{m} & =A_{a}D_{a}+\epsilon_{a} \end{array}\right.$$

Then, the mean value solution of the navigation error propagation equation for Equation (22) is

(26) $$\eta =\eta_{0}+S_{\eta g}D_{g}$$

where

(27) $$S_{\eta g}=\int_{0}^{t}C_{m}^{i}A_{g}d\tau$$

And using the basic method of variation of constant, the mean value solution of navigation error propagation equation for Equation (23) is

(28) $$\delta v^{i}=\mathrm{Exp}\left(-\left[\omega_{\mathrm{ie}}^{i}\times \right]t\right)\left\{\delta v_{0}+\int_{0}^{t}\mathrm{Exp}\left(\left[\omega_{\mathrm{ie}}^{i}\times \right]\tau \right)\left(-\left[f^{i}\times \right]\eta +C_{m}^{i}\delta f^{m}\right)d\tau \right\}$$

where Exp represents the exponential function of a matrix.

Note that $\mathrm{Exp}\left(-\left[\omega_{\mathrm{ie}}^{i}\times \right]t\right)=R_{z}\left(\omega_{e}t\right)$; thus, Equation (28) can be reduced to

(29) $$\delta v^{i}=R_{z}\left(\omega_{e}t\right)\delta v_{0}+S_{v\eta }\eta_{0}+S_{vg}D_{g}+S_{va}D_{a}$$

where

(30) $$\left\{\begin{array}{cc} S_{v\eta } & =-R_{z}\left(\omega_{e}t\right)\int_{0}^{t}R_{z}\left(-\omega_{e}\tau \right)\left[f^{i}\times \right]d\tau \\ S_{vg} & =-R_{z}\left(\omega_{e}t\right)\int_{0}^{t}R_{z}\left(-\omega_{e}\tau \right)\left[f^{i}\times \right]S_{\eta g}d\tau \\ S_{va} & =R_{z}\left(\omega_{e}t\right)\int_{0}^{t}R_{z}\left(-\omega_{e}\tau \right)C_{m}^{i}A_{a}d\tau \end{array}\right.$$

Still using the basic method of variation of constant, the mean value solution of navigation error propagation equation for Equation (23) is

(31) $$\begin{array}{cc} \delta r^{i} & =\mathrm{Exp}\left(\left[\omega_{ie}\times \right]t\right)\left\{\delta r_{0}+\int_{0}^{t}\mathrm{Exp}\left(-\left[\omega_{ie}\times \right]\tau \right)\delta v^{i}d\tau \right\}\\ & =R_{z}\left(-\omega_{e}t\right)\delta r_{0}+S_{\mathrm{rv}}\delta v_{0}+S_{r\eta }\eta_{0}+S_{rg}D_{g}+S_{ra}D_{a} \end{array}$$

Among them,

(32) $$\left\{\begin{array}{cc} S_{rv} & =R_{z}\left(-\omega_{e}t\right)\int_{0}^{t}R_{z}\left(\omega_{e}\tau \right)d\tau \\ S_{r\eta } & =R_{z}\left(-\omega_{e}t\right)\int_{0}^{t}R_{z}\left(\omega_{e}\tau \right)S_{v\eta }d\tau \\ S_{rg} & =R_{z}\left(-\omega_{e}t\right)\int_{0}^{t}R_{z}\left(\omega_{e}\tau \right)S_{vg}d\tau \\ S_{ra} & =R_{z}\left(-\omega_{e}t\right)\int_{0}^{t}R_{z}\left(\omega_{e}\tau \right)S_{va}d\tau \end{array}\right.$$

It can be seen that the navigation errors η, $\delta v^{i}$, and $\delta r^{i}$ can be written as the product of the sensitivity matrix $S_{**}$, the corresponding initial error ($\eta_{0}$, $\delta v_{0}$, and $\delta r_{0}$), and the IMU error coefficients ($D_{g}$ and $D_{a}$).

From Equations (27), (30) and (32), the sensitivity matrix depends entirely on the time histories of $f^{i}$ and $C_{m}^{i}$. Thus, it depends on the standard flight trajectory of the launch vehicle ($f^{i}$) and the RINS operation strategy ($C_{m}^{i}$).

### 2.3. RINS Operation Strategy for Launch Vehicle

**Figure 3:**
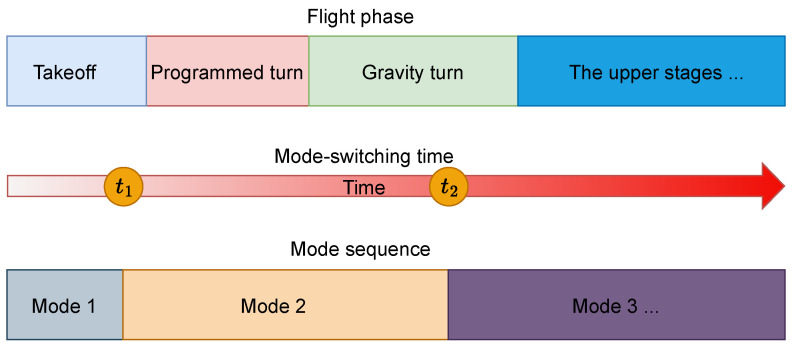
General RINS operation strategy description.

As mentioned earlier, the launch vehicle is unsuitable for the rotation modulation strategy due to the short working time. Therefore, this paper mainly studies the INS operation strategy under two navigation modes: near-platform and strap-down. Figure 3 is a schematic of each flight phase and RINS navigation mode.

Due to the short flight time, complex strategies are of little value, so this paper considers the simple strategy shown in Figure 1, namely: from the near-platform mode switch to the strap-down mode or from the strap-down mode switch to the near-platform mode. Therefore, the focus of optimization is the switching time.

Without a loss of generality, we assume a dual-axis RINS is used. The inner axis installed perpendicular to the pitching-plane is shown in Figure 1, and the outer axis is in the pitching-plane. At launch, the inner axis is parallel to the *z* axis of the measurement coordinate system, and the outer axis is parallel to the *y* axis of the measurement coordinate system.

For a period of time after takeoff, the two axes of rotation are controlled so that the attitude of the measuring coordinate system *m* with respect to the pitching-plane remains unchanged: $\int_{0}^{t}\omega_{z}=0$ and $\int_{0}^{t}\omega_{y}=0$; this is the near-platform mode. Then the switch to strap-down mode starts at a certain time.

## 3. Mode-Switching Strategy

### 3.1. Orbit Injection Error Estimation Theory

When the mode-switching strategy is adopted, the navigation indication error of the orbit injection point includes three parts:

1. The navigation error of the near-platform mode;
2. The navigation error of the switching process;
3. The navigation error of the strap-down mode.

#### 3.1.1. Switching Process Error

According to the general configuration, we assumed that the measurement coordinate system coincided with the vehicle’s body coordinate system at launch. Due to the turning motion of the vehicle’s body, when exiting the near-platform mode, the orientation of the inertial assembly will not meet the requirements of the strap-down mode so that the mode switching will execute a rotation action. Since the rotation of the projectile body generally occurs in the plane of trajectory, the rotation in the switching process is basically in that plane, which is approximately a fixed-axis rotation.

Therefore, the equivalent rotation vector of the switching process is ϕ in Equation (33).

(33) $$\phi \approx \int_{0}^{\Delta T}\omega d\tau$$

Thus, the error of the equivalent rotation vector is

(34) $$\Delta \phi \approx \int_{0}^{\Delta T}\delta \omega d\tau$$

Considering the short time, only the error caused by the linear part of the error model needs to be estimated; then,

(35) $$\Delta \phi \approx \int_{0}^{\Delta T}k_{g0}+k_{g1}\omega d\tau \approx k_{g1}\phi \left(\Delta T\to 0\right)$$

It can be seen that the error in the switching process is mainly an attitude angle increment, and its magnitude is directly proportional to the error coefficient $k_{g1}$ of the gyroscope.

Assuming that the switching time is $t_{s}$, since ϕ depends on the angle of pitch ϕ of the launch vehicle, ϕ is a function of $t_{s}$, which can be calculated by standard trajectory and denoted as

(36) $$\phi =\phi \left(t_{s}\right)$$

#### 3.1.2. Error Propagation Model with Mode-Switching Strategy

As mentioned above, assuming that the system switches from near-platform mode to strap-down mode, the RINS of the launch vehicle successively applies the near-platform mode, mode-switching process, and strap-down mode. The near-platform navigation and switching process errors are initial errors for the strap-down mode. This paper provides a quick calculation method of the sensitivity matrix when switching from near-platform to strap-down.

Assuming that time of the switching process is very short (ΔT→0), according to the estimated Formula (35), the attitude error caused by the switching process is

(37) $$\begin{array}{cc} \Delta \phi \approx k_{g1}\phi = & \left[\begin{array}{ccc} \phi^{T} & & \\ & \phi^{T} & \\ & & \phi^{T} \end{array}\right]{\bar{k}}_{g1}\\ & ={\mathrm{diag}}_{m}\left(\phi \right){\bar{k}}_{g1}\\ & =S_{g}^{\prime }\left(\phi \right)D_{g} \end{array}$$

Among them, the sensitivity matrix of the switching process is

(38) $$S_{g}^{\prime }\left(\phi \right)=\left[\begin{array}{ccc} 0_{3\times 3} & {\mathrm{diag}}_{m}\left(\phi \right) & 0_{3\times 3} \end{array}\right]$$

It is assumed that the input in near-platform mode and strap-down mode are $\omega^{p}$, $f^{p}$, and $\omega^{s}$, $f^{s}$, respectively.

According to Equations (26), (29) and (31), the error at the end time of the near-platform mode is

(39) $$\left\{\begin{array}{cc} \eta_{f}^{p} & =\eta_{0}+S_{\eta g}^{p}\left(t_{s}\right)D_{g}\\ \delta v_{f}^{p} & =\delta v_{0}+S_{v\eta }^{p}\left(t_{s}\right)\eta_{0}+S_{vg}^{p}\left(t_{s}\right)D_{g}+S_{va}^{p}\left(t_{s}\right)D_{a}\\ \delta r_{f}^{p} & =\delta r_{0}+S_{rv}^{p}\left(t_{s}\right)\delta v_{0}+S_{r\eta }^{p}\left(t_{s}\right)\eta_{0}+S_{rg}^{p}\left(t_{s}\right)D_{g}+S_{ra}^{p}\left(t_{s}\right)D_{a} \end{array}\right.$$

The attitude error generated by the switching process is

(40) $$\Delta \phi =S_{g}^{\prime }\left(\phi \left(t_{s}\right)\right)D_{g}$$

So the initial error of the strap-down mode is

(41) $$\left\{\begin{array}{cc} \eta_{0}^{s} & =\eta_{f}^{p}+\Delta \phi \\ \delta v_{0}^{s} & =\delta v_{f}^{p}\\ \delta r_{0}^{s} & =\delta r_{f}^{p} \end{array}\right.$$

According to Equations (27), (30) and (32), the sensitivity matrix in the strap-down mode are

(42) $$\left\{\begin{array}{cc} \Delta S_{\eta g}^{s} & =\int_{t_{s}}^{t}C_{m}^{i}A_{g}^{s}d\tau \\ & =S_{\eta g}^{s}\left(t\right)-S_{\eta g}^{s}\left(t_{s}\right)\\ \Delta S_{\mathrm{vg}}^{s} & =R_{z}\left(\omega_{e}t\right)\int_{t_{s}}^{t}-R_{z}\left(-\omega_{e}\tau \right)\left[f^{i}\times \right]\Delta S_{\eta g}^{s}d\tau \\ & =S_{\mathrm{vg}}^{s}\left(t\right)-S_{\mathrm{vg}}^{s}\left(t_{s}\right)-\left[S_{v\eta }^{s}\left(t\right)-S_{v\eta }^{s}\left(t_{s}\right)\right]S_{\eta g}^{s}\left(t_{s}\right)\\ \Delta S_{rg}^{s} & =R_{z}\left(-\omega_{e}t\right)\int_{t_{s}}^{t}R_{z}\left(\omega_{e}\tau \right)\Delta S_{\mathrm{vg}}^{s}d\tau \\ & =S_{rg}^{s}\left(t\right)-S_{rg}^{s}\left(t_{s}\right)-\left[S_{r\eta }^{s}\left(t\right)-S_{r\eta }^{s}\left(t_{s}\right)\right]S_{\eta g}^{s}\left(t_{s}\right)\\ & +\left[S_{rv}^{s}\left(t\right)-S_{rv}^{s}\left(t_{s}\right)\right]\left[S_{v\eta }^{s}\left(t_{s}\right)S_{\eta g}^{s}\left(t_{s}\right)-S_{vg}^{s}\left(t_{s}\right)\right]\\ \Delta S_{\mathrm{va}}^{s} & =R_{z}\left(\omega_{e}t\right)\int_{t_{s}}^{t}R_{z}\left(-\omega_{e}\tau \right)C_{m}^{i}A_{a}^{s}d\tau \\ & =S_{\mathrm{va}}^{s}\left(t\right)-S_{\mathrm{va}}^{s}\left(t_{s}\right)\\ \Delta S_{ra}^{s} & =R_{z}\left(-\omega_{e}t\right)\int_{t_{s}}^{t}R_{z}\left(\omega_{e}\tau \right)\Delta S_{\mathrm{va}}^{s}d\tau \\ & =S_{ra}^{s}\left(t\right)-S_{ra}^{s}\left(t_{s}\right)-\left[S_{rv}^{s}\left(t\right)-S_{rv}^{s}\left(t_{s}\right)\right]S_{va}^{s}\left(t_{s}\right)\\ \Delta S_{v\eta }^{s} & =R_{z}\left(\omega_{e}t\right)\int_{t_{s}}^{t}-R_{z}\left(-\omega_{e}\tau \right)\left[f^{i}\times \right]d\tau \\ & =S_{v\eta }^{s}\left(t\right)-S_{v\eta }^{s}\left(t_{s}\right)\\ \Delta S_{k}^{s} & =R_{z}\left(-\omega_{e}t\right)\int_{t_{s}}^{t}R_{z}\left(\omega_{e}\tau \right)\Delta S_{v\eta }^{s}d\tau \\ & =S_{k}^{s}\left(t\right)-S_{k}^{s}\left(t_{s}\right)-\left[S_{rv}^{s}\left(t\right)-S_{rv}^{s}\left(t_{s}\right)\right]S_{v\eta }^{s}\left(t_{s}\right)\\ \Delta S_{\mathrm{rv}}^{s} & =R_{z}\left(-\omega_{e}t\right)\int_{t_{s}}^{t}R_{z}\left(\omega_{e}\tau \right)d\tau \\ & =S_{\mathrm{rv}}^{s}\left(t\right)-S_{\mathrm{rv}}^{s}\left(t_{s}\right) \end{array}\right.$$

Then, the error to the orbit injection point $t_{k}$ is

(43) $$\left\{\begin{array}{cc} \eta_{k} & =\eta_{f}^{s}=\eta_{0}+S_{\eta g}^{k}D_{g}\\ \delta v_{k} & =\delta v_{f}^{s}=\delta v_{0}+S_{v\eta }^{k}\eta_{0}+S_{vg}^{k}D_{g}+S_{va}^{k}D_{a}\\ \delta r_{k} & =\delta r_{f}^{s}=\delta r_{0}+S_{\mathrm{rv}}^{k}\delta v_{0}+S_{k}^{k}\eta_{0}+S_{rg}^{k}D_{g}+S_{ra}^{k}D_{a} \end{array}\right.$$

In which,

(44) $$\left\{\begin{array}{cc} S_{\eta g}^{k} & =S_{\eta g}^{p}\left(t_{s}\right)+S_{g}^{\prime }\left(\phi \left(t_{s}\right)\right)+\Delta S_{\eta g}^{s}\left(t_{k}\right)\\ S_{v\eta }^{k} & =S_{v\eta }^{p}\left(t_{s}\right)+\Delta S_{v\eta }^{s}\left(t_{k}\right)\\ S_{va}^{k} & =S_{va}^{p}\left(t_{s}\right)+\Delta S_{va}^{s}\left(t_{k}\right)\\ S_{vg}^{k} & =S_{vg}^{p}\left(t_{s}\right)+\Delta S_{vg}^{s}\left(t_{k}\right)+\Delta S_{v\eta }^{s}\left(t_{k}\right)\left[S_{\eta g}^{p}\left(t_{s}\right)+S_{g}^{\prime }\left(\phi \left(t_{s}\right)\right)\right]\\ S_{\mathrm{rv}}^{k} & =S_{\mathrm{rv}}^{p}\left(t_{s}\right)+\Delta S_{\mathrm{rv}}^{s}\left(t_{k}\right)\\ S_{k}^{k} & =S_{k}^{p}\left(t_{s}\right)+\Delta S_{k}^{s}\left(t_{k}\right)+\Delta S_{\mathrm{rv}}^{s}\left(t_{k}\right)S_{v\eta }^{p}\left(t_{s}\right)\\ S_{ra}^{k} & =S_{ra}^{p}\left(t_{s}\right)+\Delta S_{\mathrm{rv}}^{s}\left(t_{k}\right)S_{va}^{p}\left(t_{s}\right)+\Delta S_{ra}^{s}\left(t_{k}\right)\\ S_{rg}^{k} & =S_{rg}^{p}\left(t_{s}\right)+\Delta S_{rg}^{s}\left(t_{k}\right)+\Delta S_{\mathrm{rv}}^{s}\left(t_{k}\right)S_{vg}^{p}\left(t_{s}\right)\\ & +\Delta S_{k}^{s}\left(t_{k}\right)\left[S_{\eta g}^{p}\left(t_{s}\right)+S_{g}^{\prime }\left(\phi \left(t_{s}\right)\right)\right] \end{array}\right.$$

Under normal launch conditions, the initial velocity errors $\delta v_{0}$ and the initial position errors $\delta r_{0}$ can be ignored. Therefore, only the initial attitude error $\eta_{0}$ is considered in this paper, which is caused by incomplete initial alignment of inertial navigation.

Thus, we can calculate the sensitivity matrix of the orbit injection point in the following way:

1. The acceleration and angular velocity time series in pure strap-down and pure near-platform modes are calculated using standard trajectories.
2. Pre-calculate $S_{**}^{s}\left(t_{i}\right)$ and $S_{**}^{p}\left(t_{i}\right)$ according to Equations (27), (30) and (32).
3. For any switch time $t_{s}$, construct sensitivity matrix $S_{**}^{k}$ of injecting point $t_{k}$ according to Equations (38), (42) and (44).

According to the above calculation process, for any switching time point $t_{s}$, the near-platform and strap-down sensitivity matrix only need to be calculated once, significantly reducing the calculation cost in practical applications.

#### 3.1.3. Distribution Estimation of Orbit Injection Error

The alignment error $\eta_{0}$ and IMU error coefficients $D_{a}$, $D_{g}$ of the RINS is unknown before the flight. Still, their distribution can be estimated after multiple missions [23]. The inertial navigation strategy designed in this paper should have the optimal average performance under this distribution. The inertial navigation error coefficient can be physically considered as a definite constant value for a short time, but because it is unknown, it can be treated as a random constant. That is, the inertial navigation error coefficient is assumed to be an independent normal distributed random quantity with zero mean and known standard deviation.

Let D be the vector composed by alignment error $\eta_{0}$ and the error coefficients of the accelerometer and gyroscope, i.e.,

(45) $$D=\left[\begin{array}{c} \eta_{0}\\ D_{a}\\ D_{g} \end{array}\right]$$

and

(46) $$\left\{\begin{array}{cc} S_{v} & =\left[\begin{array}{c} S_{v\eta }^{k}\\ S_{\mathrm{va}}^{k}\\ S_{\mathrm{vg}}^{k} \end{array}\right]\\ S_{r} & =\left[\begin{array}{c} S_{r\eta }^{k}\\ S_{\mathrm{ra}}^{k}\\ S_{\mathrm{rg}}^{k} \end{array}\right] \end{array}\right.$$

In this way, the navigation error estimation of the orbit injection point is

(47) $$\left\{\begin{array}{cc} \delta v^{k} & =S_{v}D\\ \delta r^{k} & =S_{r}D \end{array}\right.$$

So the covariance of error of the orbit injection point is

(48) $$\left\{\begin{array}{cc} \Sigma_{\delta r} & =E\left(S_{r}DD^{T}S_{r}\right)=S_{r}^{k}\Sigma_{D}S_{r}^{T}\\ \Sigma_{\delta v} & =E\left(S_{v}DD^{T}S_{v}\right)=S_{v}\Sigma_{D}S_{v}^{T}\\ \Sigma_{\delta rv} & =E\left(S_{r}DD^{T}S_{v}\right)=S_{r}\Sigma_{D}S_{v}^{T} \end{array}\right.$$

where $\Sigma_{D}$ is the covariance matrix of each error coefficient. Assuming that the error coefficients are independent of each other, it is

(49) $$\Sigma_{D}=\left[\begin{array}{cccc} \sigma_{1}^{2} & & & \\ & \sigma_{2}^{2} & & \\ & & \ddots & \\ & & & \sigma_{n}^{2} \end{array}\right]$$

According to the multivariate normal distribution theory, the probability distribution of $\delta v^{h}$ ($\delta r^{e}$) is

(50) $$N\left(x,\mu ,\Sigma \right)=\frac{1}{\left(2\pi \right)n^{2}{\left|\Sigma \right|}^{1/2}}\exp\left[-\frac{1}{2}{\left(x-\mu \right)}^{T}\Sigma^{-1}\left(x-\mu \right)\right]$$

And further, the corresponding distribution function is

(51) $$P\left[{\left(x-\mu \right)}^{T}\Sigma^{-1}\left(x-\mu \right)\leq c^{2}\right]=1-\frac{2\Gamma \left(\frac{3}{2},\;\frac{c^{2}}{2}\right)}{\sqrt{\pi }}$$

where Γ represents the incomplete Gamma function and is defined as

(52) $$\Gamma \left(a,z\right)=\int_{z}^{\infty }t^{a-1}e^{-t}dt$$

Note that ${\left(x-\mu \right)}^{T}\Sigma^{-1}\left(x-\mu \right)\leq c^{2}$ is an ellipsoid, the meaning of the above equation is the probability that the error falls inside the ellipsoid, and the three-axis radius of the ellipsoid is $\Lambda^{1/2}c,\Lambda =\mathrm{diag}\left(\lambda_{1},\lambda_{2},\lambda_{3}\right)$, which is a diagonal matrix formed by the eigenvalues of the covariance matrix Σ.

**Figure 4:**
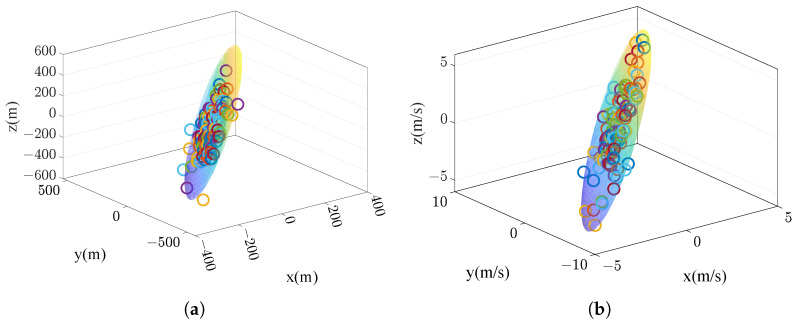
Position and velocity error distribution prediction. (**a**) Position error distribution prediction. (**b**) Velocity error distribution prediction.

We executed a Monte Carlo simulation to verify the distribution function Equation (51). This simulation randomly generated 100 groups of alignment error and IMU error coefficients and compared the navigation error by a standard navigation calculation with the probability ellipsoid provided by the error propagation model. The results are shown in Figure 4a,b, in which the shaded area is the probability ellipsoid (about 95.4% confidence). From the figure, it is shown that the Monte Carlo simulation results are in good agreement with the prediction results based on the sensitivity matrix, which fully proves the correctness of the error distribution estimation model.

### 3.2. Generate Strategy by Optimization

Among the conventional six orbital roots, the argument of periapsis does not apply to the near-circular orbit, and the true perigee angle indicates the position of the satellite in the orbit rather than the orbit itself, so they are not included in the comparative analysis. Thus, in this paper, orbit shape parameters (semi-major axis *a* and eccentricity *e*) and orbit plane position parameters (orbital inclination *i* and right ascension Ω) are mainly considered. They are

(53) $$\left\{\begin{array}{cccc} a & =\frac{r}{2-\frac{rv^{2}}{\mu }},\; & e & =\sqrt{1-\frac{h^{2}}{\mu a}}\\ i & =\arccos\frac{h_{z}}{h},\; & \Omega & =\arctan\frac{-h_{x}}{h_{y}} \end{array}\right.$$

where angular momentum h=r×v and h=∥h∥, $\mu =3.9860e+14m^{3}/s^{2}$ is the gravitational constant of the Earth.

In view of Equation (53), *a*, *e*, *i*, and Ω are all functions of the position of the orbital injection point $r_{k}$ and the velocity $v_{k}$.

Without a loss of generality, we assume that the target orbit parameter *p* is a function of the position of the orbital injection point $r_{k}$ and the velocity $v_{k}$.

Notice that

(54) $$\delta p\left(r_{k},\;v_{k}\right)={\left(\frac{\partial p}{\partial r_{k}}\right)}^{T}\delta r_{k}+{\left(\frac{\partial p}{\partial v_{k}}\right)}^{T}\delta v_{k}$$

Therefore, the corresponding variance is

(55) $$\begin{array}{cc} \mathrm{var}\left(\delta p\right)=E\left({\left(\delta p\left(r_{k},\;v_{k}\right)\right)}^{2}\right) & ={\left(\frac{\partial p}{\partial r_{k}}\right)}^{T}\Sigma_{\delta r}\left(\frac{\partial p}{\partial r_{k}}\right)\\ & +{\left(\frac{\partial p}{\partial v_{k}}\right)}^{T}\Sigma_{\delta v}\left(\frac{\partial p}{\partial v_{k}}\right)\\ & +2{\left(\frac{\partial p}{\partial r_{k}}\right)}^{T}\Sigma_{\delta rv}\left(\frac{\partial p}{\partial v_{k}}\right) \end{array}$$

Then, the optimal strategy can be obtained by the following optimization problem

(56) $$\min_{t_{s}}\mathrm{std}\left(\delta p\right)=\sqrt{\mathrm{var}\left(\delta p\right)}$$

According to Equations (54)–(56), we can obtain the corresponding variance calculation and optimization methods of *a*, *e*, *i*, Ω by specifying *p* as *a*, *e*, *i*, Ω, respectively.

According to the error estimation theory in Section 3 and Equations (54)–(56), users can optimize any representable indicator of $p\left(r_{k},\;v_{k}\right)$ to obtain a switching time that minimizes the standard deviation of its error. For example, for p=a, we can then calculate the change curve of std(δa) with the switching time and use the time corresponding to the lowest point of the curve as the switching time.

## 4. Numerical and Semi-Physical Experiments

### 4.1. Numerical Experiment

#### 4.1.1. Launch Mission and Inertial Navigation Configuration

Consider a two-stage launch vehicle (Falcon 9 [24,25]); the main parameters of each stage are shown in Table 1. The corresponding IMU parameters are shown in Table 2.

Due to the incomplete alignment of the launch vehicle before launch, the initial attitude error is assumed to be distributed as Table 3. Since the velocity and position at launch time is known, the initial errors in velocity and position are ignored. The technique proposed in this paper is mainly used to suppress instrument errors. Therefore, we assume a high alignment accuracy. Considering the challenge of achieving such alignment accuracy in practical applications, we discuss the role of the proposed method when alignment accuracy decreases in Section 5.

**Figure 5:**
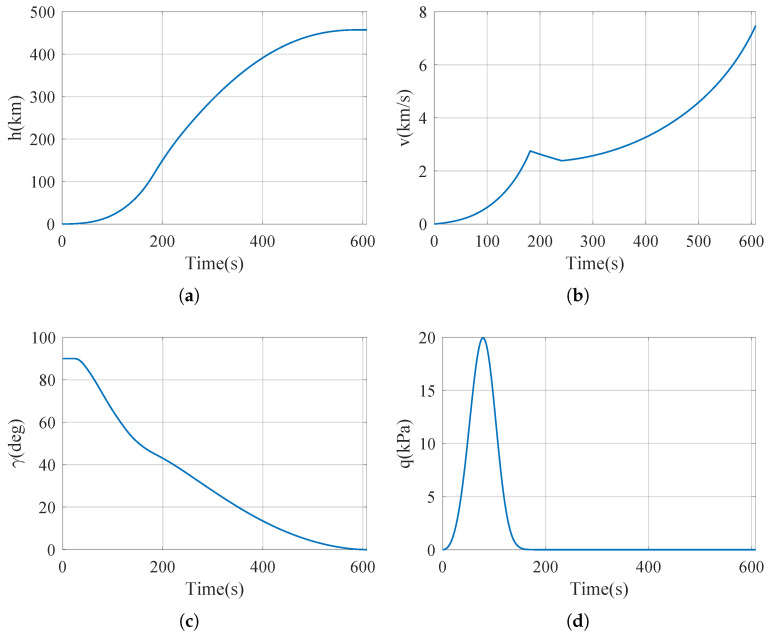

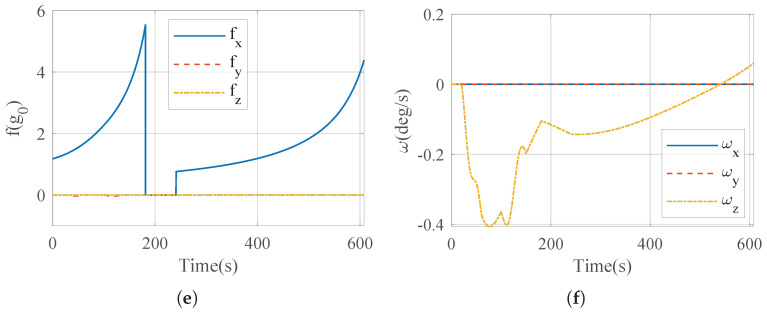
Trajectory of the launch vehicle. (**a**) Height curve. (**b**) Velocity curve. (**c**) Flight path angle curve. (**d**) Dynamic pressure curve. (**e**) Apparent acceleration curve. (**f**) Angular velocity curve.

The flight trajectories are shown in Figure 5.

**Figure 6:**
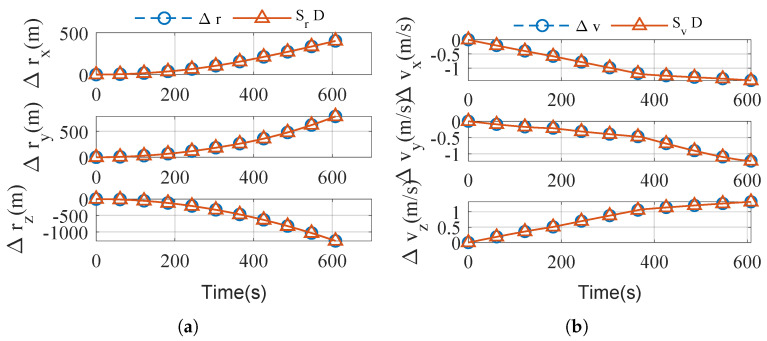
Verification of the navigation error estimation. (**a**) Δr comparison. (**b**) Δv comparison.

Using flight trajectories in Figure 5 and IMU parameters in Table 2, let t_*s*_ = 300 s. The accuracy of the error estimation equation, Equation (47), can be verified by comparing the actual navigation calculation with the theoretical estimation Equation (47), see Figure 6.

#### 4.1.2. Orbit Injection Error Estimation

According to the error propagation model with the mode-switching strategy in Section 3.1.2 and Equation (56), we can calculate the impact of the switching time $t_{s}\in \left[0,\;t_{k}\right]$ on the orbit error in a computationally cheap way.

**Figure 7:**
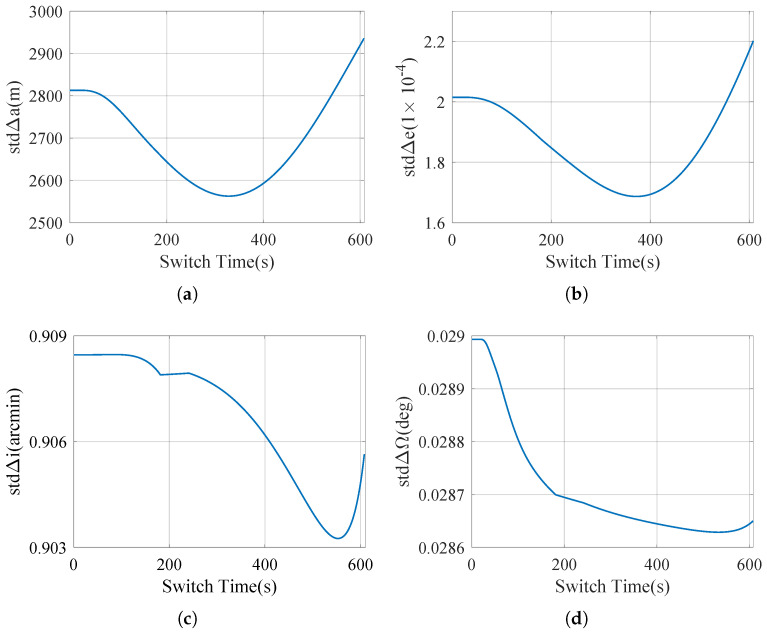
Orbit error of the rocket. (**a**) Semi-major axis error with the switching time. (**b**) Eccentricity error with the switching time. (**c**) Orbital inclination error with the switching time. (**d**) Right ascension of the ascending node error with the switching time.

Figure 7 shows the change of major orbital root error with switching time, in which *a* represents the semi-major axis, *e* represents the eccentricity, *i* represents the orbital inclination, and Ω represents the right ascension of the ascending node.

Note that the left and right endpoints of the curve represent the strap-down mode and near-platform mode, respectively.

According to Figure 7, users can choose when to switch based on the task metrics they care about. For example, if you want to improve the accuracy of the orbit’s semi-major axis, then selecting *t_s_* = 367 s corresponding to the lowest point of the curve based on Figure 7a is the required switching time. In view of Equation (54), if other task indicators other than *a*, *e*, *i*, Ω are required, then the corresponding $p\left(r_{k},\;v_{k}\right)$ can be constructed, and the required switching time can be selected according to the corresponding curve.

As mentioned earlier, since there is no need to recalculate the sensitivity matrix for each different switching time, the computation of the objective function is computationally cheap. When the time step is ten milliseconds, it takes about 1.3 s on an ordinary laptop computer (Intel i7-1185G7 3.00 GHz with 16 GB RAM) to generate a full optimization curve (Figure 7), which means that the traversal method can be directly used for optimization.

From Figure 7a, it can be seen that at the switch time *t_s_* = 367 s, the semi-major axis error decreases 14% compared with the near-platform mode (decreases 10% compared with the strap-down mode). The other orbit errors change only a little with the switch time because they are kept small.

#### 4.1.3. Monte Carlo Simulation

In order to verify the optimization results in the previous section, a Monte Carlo simulation was used to perform numerical navigation verification experiments. Inputs to the inertial navigation system are still generated by the flight trajectory, Figure 5. In the simulation, the initial attitude error caused by incomplete initial alignment in the inertial navigation system of the launch vehicle before launch is considered, and its distribution parameters are shown in Table 3. In the simulation, the error characteristics of the accelerometer and gyroscope are still generated randomly according to Table 2.

**Figure 8:**
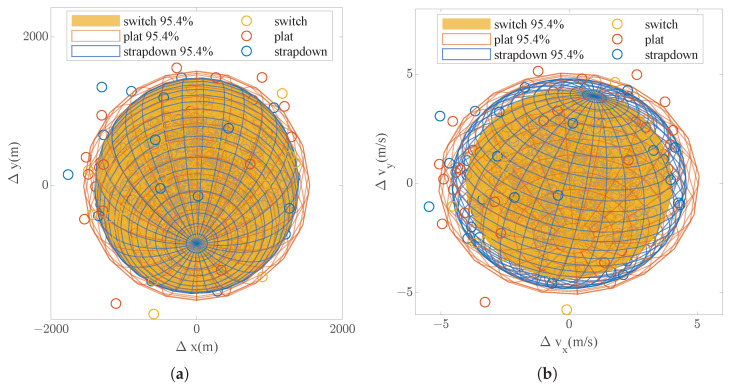
Monte Carlo simulation results. (**a**) Position error distribution. (**b**) Velocity error distribution.

The results of the 500 repeated simulations are shown in Figure 8, where the shaded area represents the error ellipsoid (about 95.4% confidence). It can be seen that the position error and speed error through mode switching are better than the pure strap-down and near-platform mode; the speed error, especially, has been significantly improved.

**Figure 9:**
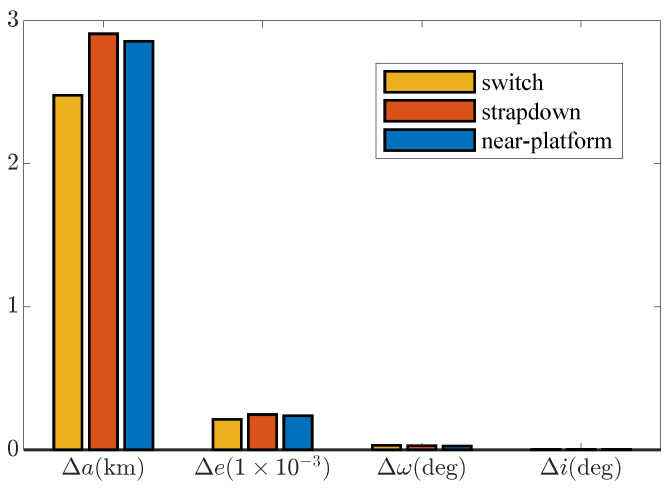
Monte Carlo orbit error.

Figure 9 evaluates the effect of mode switching from the perspective of the orbit parameter error. It can be seen that the effect of mode switching is mainly reflected in the improvement of the orbit semi-major axis accuracy. Among them, the switching strategy improves the accuracy of the orbit semi-major axis by about 13.3% compared with the strap-down mode and improves by about 15.1% compared with the near-platform mode. This is a nice improvement on a simple strategy.

### 4.2. Semi-Physical Experiment

**Figure 10:**
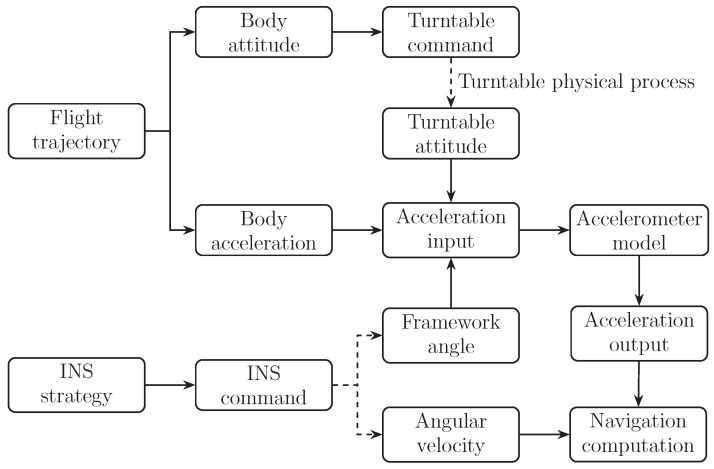
Principle of the turntable experiment.

In order to further verify the effectiveness of the mode-switching strategy, this paper conducted a semi-physical experiment. The schematics of the experimental system are shown in Figure 10.

**Figure 11:**
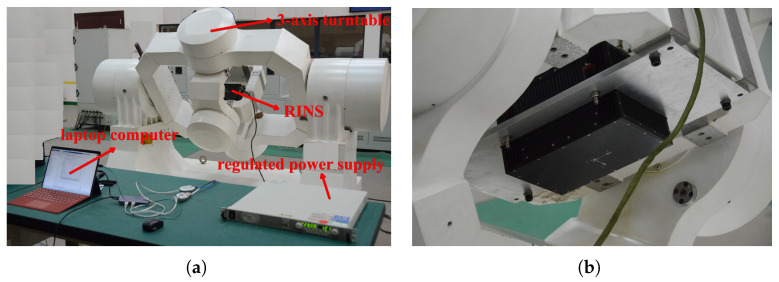
Experimental system. (**a**) Experimental system connect. (**b**) INS installation in the turntable.

As shown in Figure 11, the experimental system consists of a 3-axis turntable, a RINS, a regulated power supply, and a laptop computer. Among them, the 3-axis turntable is used to simulate the attitude change during the rocket launch, the RINS is used to measure the attitude change, the regulated power supply is used to power the RINS, and the laptop is used for data acquisition and processing. The flight trajectory and inertial navigation strategy used in the experiment were consistent with Section 4.1.

**Figure 12:**
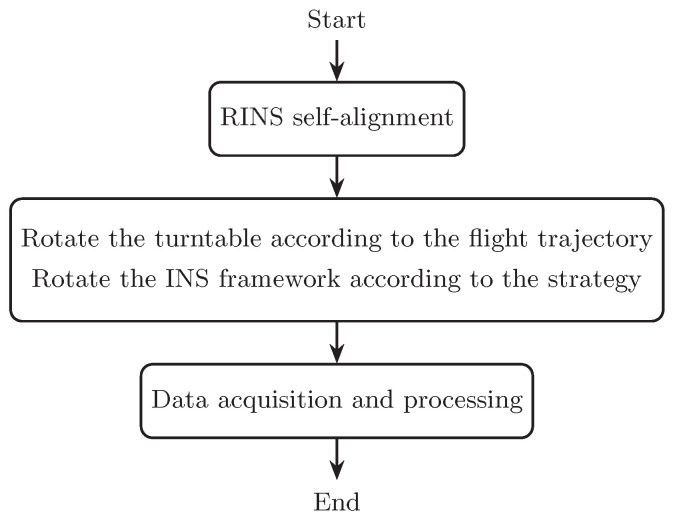
Experimental process.

**Figure 13:**
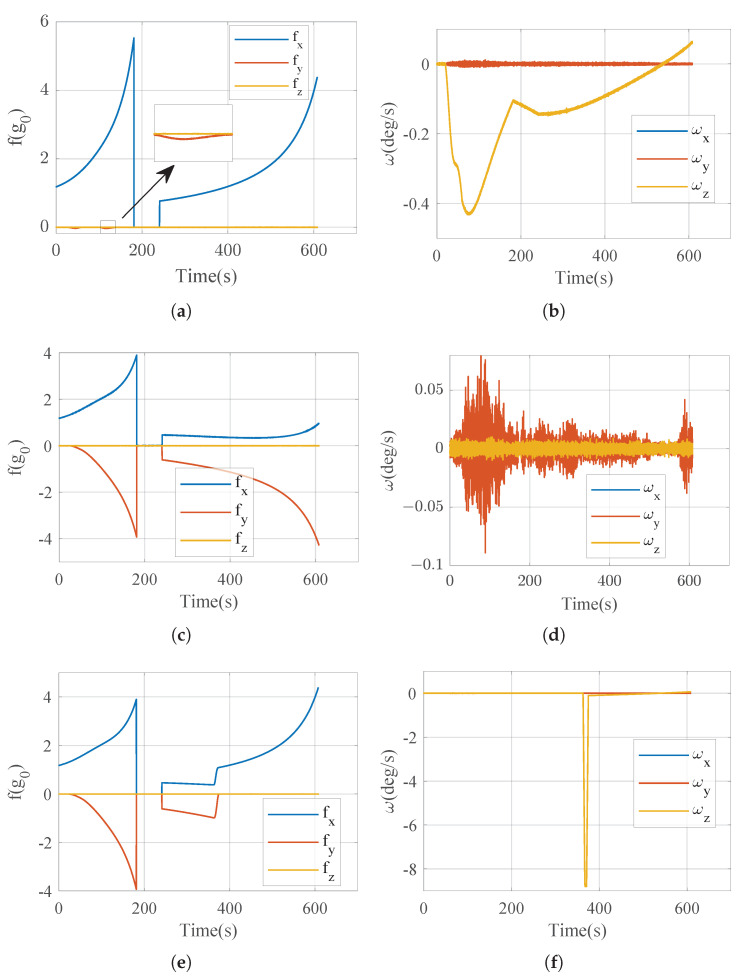
Turntable frame angle. (**a**) Acceleration output in strap-down mode. (**b**) Angular velocity output in strap-down mode. (**c**) Acceleration output in near-platform mode. (**d**) Angular velocity output in near-platform mode. (**e**) Acceleration output in mode-switching strategy. (**f**) Angular velocity output in mode-switching strategy.

The experiment process is shown in Figure 12. Ten experiments were carried out for the strap-down mode, near-platform mode, and switching strategy, and the navigation error was analyzed. Figure 13 is the IMU output in some experiments. Table 4 shows the statistical results of navigation errors in all experiments.

The switching mode’s speed and position accuracy are improved compared with the strap-down mode and near-platform mode, and the orbit injection accuracy is mainly reflected in the semi-major axis accuracy. Compared with the strap-down mode, the orbit semi-major axis accuracy of the switching strategy is improved by about 12.4% (compared with the mean value), and compared with the near-platform mode, the orbit semi-major axis accuracy is improved by about 25.7% (compared with the mean value), which is a good improvement for such a simple strategy. At the same time, the variance of the orbit semi-major error is also smaller than the pure near-platform or strap-down mode.

## 5. Discussion

There are challenges in considering the precision required for Table 3 in Section 4. Therefore, here, we discuss how the effect of the proposed method will change when the alignment accuracy is reduced. For this reason, we assume that the alignment accuracy is Table 5.

**Figure 14:**
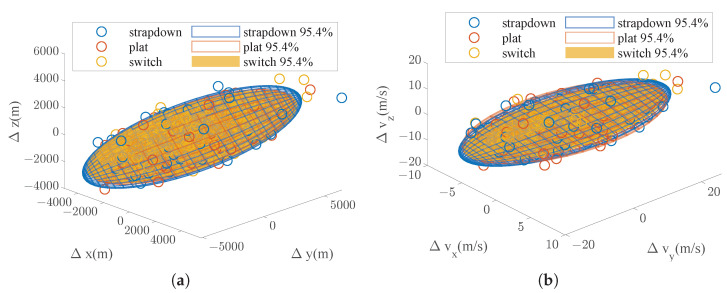
Monte Carlo simulation results under bigger initial errors. (**a**) Position error distribution. (**b**) Velocity error distribution.

The new results of the 500 repeated simulations are shown in Figure 14. It can be seen that the position error and speed error through mode switching are still better than the pure strap-down and near-platform modes.

**Figure 15:**
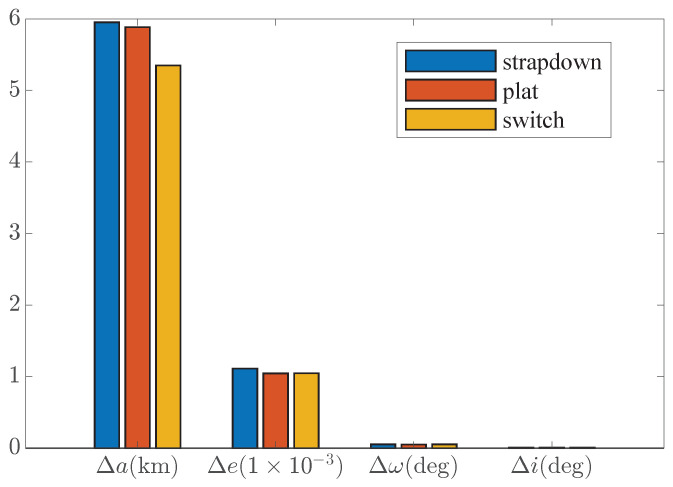
Monte Carlo orbit error under bigger initial errors.

Figure 15 evaluates the effect of mode switching from the perspective of the orbit parameter error. It can be seen that the effect of mode switching is mainly reflected in the improvement of tje orbit semi-major axis accuracy. Among them, the switching strategy improves the accuracy of the orbit semi-major axis by about 10.1% compared with the strap-down mode and improves by about 9.1% compared with the near-platform mode.

These results are significantly lower than those reported in Figure 15 in Section 4. The above results are understandable, considering that the influence of alignment accuracy increases and the influence of device accuracy on total accuracy decreases correspondingly.

The technique proposed in this paper is mainly used to suppress instrument errors. Therefore, like all methods to suppress device accuracy, the proposed method requires a good alignment accuracy, but it still achieves the goal of suppressing device error.

## 6. Conclusions

This paper introduces a mode-switching strategy for the rotational inertial navigation system to improve the launch vehicle’s orbit injection accuracy. This method reduces the accumulation of navigation errors by making the inertial navigation system adopt different navigation modes, such as near-platform or strap-down, in various periods.

This paper develops a fast calculation method for the error sensitivity matrix along a flight trajectory to achieve this. Finally, the feasibility of this strategy is analyzed by a numerical simulation and turntable experiment of the Falcon 9 launch mission. The results show that the method can improve speed and position accuracy. Thus, when the guidance system is working correctly, the accuracy of the orbit injection can be improved. The method proposed in this paper only needs to optimize the timing of navigation mode switching and is easy to implement in engineering, no other cost needed. Based on the calculation and optimization theory in this paper, it will be possible to develop effective strategies for other inertial navigation systems and flight trajectories in the future.

The main limitation of the proposed method is that it requires high alignment accuracy, which is inevitable because the technique proposed in this paper is mainly used to suppress instrument errors. When the alignment accuracy decreases, although the instrument errors can still be suppressed using the method proposed in this paper, the contribution to the final navigation accuracy is reduced. Nevertheless, it still achieves the goal of suppressing device error.

## Figures and Tables

**Table 1:** The main parameters of the launch vehicle.

Parameters	First Stage	Second Stage
Mean thrust	8227 kN	934 kN
Structural weight	22,200 kg	4000 kg
Fuel weight	410,843 kg	107,509 kg
Burning time	162 s	397 s
Specific impulse	3244 m/s	3449 m/s

**Table 2:** IMU error coefficients generator.

Item	acc (1σ)	gyr (1σ)
bias	3 × 10^−4^ g_0_	1 × 10^−2^ deg/h
scale	5 ppm	5 ppm
installation	$5^{\prime \prime }$	$5^{\prime \prime }$
asymmetry	1 ppm	—
quadratic	1 × 10^−5^ g_0_^−1^	1 × 10^−5^ s/deg
noise	$1\;\times \;10^{-5}g_{0}/\sqrt{Hz}$	$1\;\times \;10^{-3}deg/h/\sqrt{Hz}$

**Table 3:** Statistical distribution of initial errors.

Item	Mean Value	Standard Deviation
$\phi_{e}$	0	$10^{\prime \prime }$
$\phi_{n}$	0	$10^{\prime \prime }$
$\phi_{u}$	0	$30^{\prime \prime }$

**Table 4:** Experiment result statistics: mean (std) of ten times.

Item/Mode	Strap-Down	Plat	Mode-Switching
$v_{x}$ (m/s)	0.591 (1.629)	−0.590 (1.660)	−0.353 (1.117)
$v_{y}$ (m/s)	−0.570 (1.344)	−0.046 (1.269)	−0.225 (1.158)
$v_{z}$ (m/s)	−0.289 (1.978)	−0.106 (1.460)	−0.246 (1.480)
*x* (m)	112.190 (428.340)	−187.040 (514.320)	−161.580 (441.620)
*y* (m)	−193.450 (448.470)	−5.759 (375.280)	−28.355 (357.430)
*z* (m)	−96.980 (607.950)	−43.214 (455.480)	−58.635 (456.150)
*a* (m)	−782.330 (2452.200)	923.370 (2590.600)	685.870 (1976.500)
*e* (1 × $10^{-3}$)	−0.031 (0.167)	−0.052 (0.215)	−0.065 (0.184)
Ω (arcmin)	0.039 (1.772)	−0.090 (1.366)	−0.116 (1.367)
*i* (arcmin)	−0.004 (0.184)	0.003 (0.138)	0.009 (0.139)

**Table 5:** Statistical distribution of bigger initial errors.

Item	Mean Value	Standard Deviation
$\phi_{e}$	0	1 archmin
$\phi_{n}$	0	1 archmin
$\phi_{u}$	0	3 archmin

## Data Availability

The raw data supporting the conclusions of this article will be made available by the authors on request.
